# Incidence of left atrial abnormalities under treatment with dabigatran, rivaroxaban, and vitamin K antagonists

**DOI:** 10.1186/s40001-016-0235-8

**Published:** 2016-10-21

**Authors:** Stefan Reers, Tolga Agdirlioglu, Michael Kellner, Matthias Borowski, Holger Thiele, Johannes Waltenberger, Michael Reppel

**Affiliations:** 1 Department of Cardiovascular Medicine, University Hospital Muenster, 48149 Muenster, Germany; 2 Department of Cardiology/Angiology/Intensive Care Medicine, University Heart Center Luebeck, Luebeck, Germany; 3 Institute of Biostatistics and Clinical Research, University of Muenster, Muenster, Germany

**Keywords:** Vitamin K antagonist, Dabigatran, Rivaroxaban, Thrombus, Atrial fibrillation, Left atrial appendage

## Abstract

**Background:**

Non-vitamin K antagonist oral anticoagulants (NOACs) such as dabigatran or rivaroxaban are alternatives to vitamin K antagonists (VKAs) for prevention of stroke and systemic embolism in patients with atrial fibrillation (AF) and atrial flutter (AFL). Incidences of risk factors for left atrium (LA) and left atrial appendage (LAA) thrombus formation, such as dense spontaneous echo contrast (SEC), low LAA velocity (LAAV) <20 cm/s under treatment with dabigatran and rivaroxaban in comparison with VKAs are unknown.

**Methods:**

We studied 306 patients with AF (94 %) and AFL (6 %) undergoing transesophageal echocardiography. Patients received VKAs (*n* = 138), dabigatran (*n* = 68), or rivaroxaban (*n* = 100) for at least 3 weeks prior to investigation. Time in therapeutic range was 67 % for VKA. Mean CHADS_2_ score and CHA_2_DS_2_-VASc score were 1.3 and 2.5, respectively. Left atrial abnormality was defined as either dense SEC, low LAAV <20 cm/s, or thrombus.

**Results:**

Any LA abnormality occurred in 9, 3, and 5 % of patients receiving VKA, dabigatran, and rivaroxaban, respectively. The most frequent abnormality was LAA thrombus (VKA: 4 %, dabigatran: 0 %, rivaroxaban: 2 %) and low LAAV of less than 20 cm/s (VKA: 4 %, dabigatran: 1 %, rivaroxaban: 1 %), followed by dense SEC (VKA: 2 %, dabigatran: 1 %, rivaroxaban: 2 %). Results of uni- and multivariate analyses revealed a numerically lower but not significantly different frequency of any LA abnormality under dabigatran (OR 0.4, 95 % Cl 0.08 − 1.88, *p* = 0.25) and rivaroxaban (OR 0.65, 95 % Cl 0.22 − 1.98, *p* = 0.45) compared to VKA.

**Conclusion:**

With respect to the incidence of LA abnormalities, dabigatran and rivaroxaban are not inferior to VKA.

## Background

Non-valvular atrial fibrillation (AF) is the most common sustained cardiac arrhythmia, and is associated not only with an increased risk of stroke and other systemic embolism but also increased mortality and morbidity [1–4]. In comparison to the general population, the risk of stroke is four- to fivefold higher in patients with AF [5]. An overview of five randomized trials has shown that treatment with vitamin K antagonists (VKA) reduced the incidence of ischemic stroke from 4.5 to 1.4 %/year with a relative risk reduction of 68 % in AF patients [6]. Pharmacokinetics and pharmacodynamics of VKA are influenced by many factors including nutrition leading to fluctuant international normalized ratio (INR) values and individual changes of the time in therapeutic range (TTR). Non-vitamin K antagonist oral anticoagulants (NOACs), including the direct thrombin inhibitor dabigatran and the factor Xa inhibitors rivaroxaban, revealed a clinical benefit compared with VKA in large-scale phase III studies [7, 8]. Despite these benefits, there are many open questions in terms of specific clinical situations. Based on guidelines, for patients with AF of >48 h duration, therapeutic anticoagulation is recommended for at least 3 weeks prior to and 4 weeks after cardioversion to exclude left atrial appendage (LAA) thrombus [9, 10]. While the efficacy of VKAs for prevention of stroke and systemic embolism in the cardioversion setting has been studied in detail [11, 12], only few studies have been published for NOACs [13–15]. A recently published study compared NOACs and VKAs in a high-risk population, i.e., patients with a median CHA_2_DS_2_-VASc score of 4. The prevalence of intracardiac thrombi under VKAs was unexpectedly high (17.8 %) bearing risk of thromboembolic events despite sufficient anticoagulation. However, it was significantly higher than under dabigatran (3.8 %) or rivaroxaban (4.1 %). The TTR of VKA patients in that study is not known. The authors concluded that the prevalence of intracardiac thrombi was lower under NOAC therapy than under VKAs [16].

Potential risk factors for thrombus formation in LA/LAA, such as dense spontaneous echo contrast (SEC), and low left atrial appendage velocity (LAAV <20 cm/s) that are known as independent risk factors for thromboembolic events have not been studied yet in detail [17–19]. It has been demonstrated that these transesophageal echocardiogram (TEE) findings were stronger predictors of thromboembolic events than the CHADS_2_ score [20] and that the annual rate of cerebral embolism was 22 %, despite oral anticoagulation [21]. Although VKAs seem to be similarly effective than NOACs, the incidences of these echocardiographic LA abnormalities under treatment with dabigatran, rivaroxaban, and VKAs especially in a mid- or low-risk population are unknown. Therefore, the aim of the present registry study was to identify the frequencies of LA abnormalities in patients treated with VKAs at a sufficient TTR of at least 65 %, dabigatran or rivaroxaban.

## Methods

### Study population

We studied 306 patients with AF and atrial flutter (AFL) under treatment with VKAs, dabigatran, or rivaroxaban referred to our department for cardioversion or catheter ablation between October 2011 and December 2014. Exclusion criteria were atrial fibrillation due to a reversible cause (e.g., hyperthyroidism, infection, transient perioperative AF), moderate or severe heart-valve disorder or prosthetic heart valve, heart transplant, need for aspirin at a dose of >100 mg/day or for both aspirin and a P_2_Y_12_ inhibitor, active liver disease, calculated creatinine clearance of <15 ml per minute, pregnancy, stroke within 14 days prior to admission, and non-compliance with drug therapy.

All included patients with creatinine clearance ≥50 ml/min and HAS-BLED score <3 in the dabigatran and rivaroxaban group received the standard dose of 150 mg BID and 20 mg OD, respectively. Only patients with moderate renal impairment (creatinine clearance 30–49 ml/min) or high bleeding risk (HAS-BLED score ≥3) were treated with reduced dose of 110 mg BID dabigatran or 15 mg OD rivaroxaban, as recommended in the European Society of Cardiology (ESC) guidelines for the management of AF [9].

### Scores

The risk of stroke or systemic embolism and hemorrhage was determined using the CHADS_2_, CHA_2_DS_2_-VASc, and HAS-BLED scores [9].

### Transthoracic (TTE) and transesophageal echocardiography (TEE)

All included patients were studied by TEE and optionally TTE (Vivid E9, GE Healthcare, USA). Echocardiographic measurements were performed according to the recommendations of the international standard practice guidelines [22, 23]. TTE was performed using a 1.5–4.6 MHz imaging transducer (M5S-D and M5Sc-D, GE Healthcare, USA). For TEE 3.0–8.0 MHz, a multiplane phased array transducer (6VT-D, GE Heathcare, USA) was used. Patients were sedated with midazolam and/or propofol. Blood pressure, heart rate, and oxygen saturation were continuously monitored. LA and LAA were studied in multiple planes. LAAs were categorized into non-banded and banded LAAs and four different morphology subgroups, as first described by Wang et al. [24]: chicken wing, wind sock, cauliflower, and cactus.

### LA abnormality

LA abnormality was defined as thrombus in LA/LAA, dense SEC, and severely reduced LAAV (≤20 cm/s) [17, 19]. We constituted a rank order: patients with proof of LA/LAA thrombus were not included in the dense SEC- or LAAV <20 cm/s group and patients with dense SEC were not included in the LAAV <20 cm/s group.

Thrombus was defined as an iso- or hyperechogenic non-muscular and non-endocardial mass detected by more than one plane axis [25]. LAAV was measured by pulsed-wave (PW) Doppler as maximal flow velocity in the proximal third of the appendage. The view with the optimal alignment was determined by color flow imaging [26]. The density of SEC was graded in 5 levels, as described before [26]: (0) absence of echogenicity, (+1) mild (minimal echogenicity located only in a part of the LA cavity, only transiently during the cardiac cycle, and only at high gain setting), (+2) mild to moderate (denser swirling than mild SEC but only demonstrable at high gain setting), (+3) moderate (dense swirling in the LAA und partially in the LA cavity with varying intensity), (+4) dense (intense echodensity and very slow swirling in LAA).

To calculate the time in therapeutic range (TTR) for VKA group patients, the Rosendaal method was used [27]. The INR values of the last 6 weeks were included in the calculation.

### Statistical analysis

The study population was described by the standard descriptive statistics such as frequencies, mean ± standard deviation, median with the corresponding interquartile range, wherever appropriate. Inferential statistics were intended to be exploratory (hypotheses generating), not confirmatory, and are interpreted accordingly. Thus, p values are to be interpreted in Fisher’s sense, representing the metric weight of evidence against the respective null hypothesis. Neither a global significance level nor local levels were determined. A *p* value ≤0.05 was considered statistically significant.

Pair-wise differences between the anticoagulation groups (VKA vs. dabigatran, VKA vs. rivaroxaban, and dabigatran vs. rivaroxaban) were assessed respectively using Fisher’s exact test (for categorical variables with two categories), the Chi^2^-test (for categorical variables with three or more categories), and the Mann–Whitney-U-test (for continuous variables).

In order to prevent biased results due to imbalanced patient’s baseline characteristics, the impact of anticoagulation on the risk of any LA/LAA abnormality was assessed by multivariate logistic regression. Model building was carried out by means of all-subset variable selection based on Akaike’s information criterion [28]. Coefficients from logistic regression were checked by the Wald-test, and odds ratios (OR) and referring 95 % confidence intervals (CI) were derived. All statistical computations were carried out using R 3.1.2 (R Core Team, 2014).

## Results

### Baseline characteristics

Table 1 shows the clinical baseline characteristics of the 306 patients included. The mean age was 67 with an interquartile range of 58–73, 60 % were male. Most of the patients had paroxysmal AF (56 %), followed by persistent AF (36 %), permanent (1 %), and longstanding persistent (<1 %). 6 % were suffering from AFL. In the VKA group, the TTR, defined as an INR between 2.0 to 3.0, was 67 %. Between the three treatment groups, some significant differences were observed (for more details see Table 1).

**Table 1:** Baseline characteristics of the study population

Variable	Dabigatran, *N* = 68 (22 %)		VKA, *N* = 138 (45 %)		Rivaroxaban, *N* = 100 (33 %)		*p* value for difference between VKA and dabigatran	*p* value for difference between VKA and rivaroxaban	*p* value for difference between dabigatran and rivaroxaban
Age: year									
Median	66		68.5		64		0.16	0.052	0.738
Interquartile range	58–72		60–74		55.8–72				
Male sex: no. (%)	48 (71 %)		78 (57 %)		57 (57 %)		0.068	1	0.078
Body–mass index									
Median	27.2		27.5		28.4		0.809	0.196	0.209
Interquartile range	24.9–31.4		25.0–30.9		25.5–32.9				
Hypertension	43 (63 %)		102 (74 %)		69 (69 %)		0.119	0.562	0.266
Diabetes mellitus	14 (21 %)		32 (23 %)		18 (18 %)		1	0.266	0.279
LA size: cm^2^									
Median	4.1		4		4.1		0.122	0.846	0.216
Interquartile range	3.6–4.5		3.4–4.3		3.1–4.4				
LA size ≥ 5.5 cm^2^: no. (%)	2 (3 %)		3 (2 %)		2 (2 %)		1	1	1
LVEF classification: no. (%)									
Normal (≥55 %)	55 (81 %)		119 (86 %)		77 (77 %)		0.567	0.328	0.652
Mildly reduced (45–54 %)	7 (10 %)		11 (8 %)		14 (14 %)				
Moderately reduced (30–44 %)	4 (6 %)		7 (5 %)		8 (8 %)				
Severely reduced (<30 %)	2 (3 %)		1 (1 %)		1 (1 %)				
Renal function; creatinine clearance; classification—no. (%)									
Normal (>80 ml/min)	57 (84 %)		102 (74 %)		74 (74 %)		0.247	0.973	0.266
Mild impairment (51–80 ml/min)	11 (16 %)		35 (25 %)		25 (25 %)				
Moderate impairment (31–50 ml/min)	0 (0 %)		1 (1 %)		1 (1 %)				
Severe impairment (≤30 ml/min)	0 (0 %)		0 (0 %)		0 (0 %)				
Classification of atrial fibrillation—no. (%)									
Paroxysmal	44 (65 %)		81 (59 %)		47 (47 %)		0.792	0.054	0.097
Persistent	20 (29 %)		49 (36 %)		42 (42 %)				
Longstanding persistent	0 (0 %)		1 (1 %)		1 (1 %)				
Permanent	1 (1 %)		3 (2 %)		0 (0 %)				
Atrial flutter	3 (4 %)		4 (3 %)		10 (10 %)				
CHADS_2_									
Mean score (±SD)	1.1 (±0.8)		1.5 (±0.9)		1.2 (±0.8)		*0.003*	*0.006*	0.521
Score—no. (%)									
0 or 1	50 (74 %)		76 (55 %)		70 (70 %)		*0.029*	*0.012*	0.647
2	14 (21 %)		42 (30 %)		26 (26 %)				
≥3	4 (6 %)		20 (14 %)		4 (4 %)				
CHA_2_DS_2_-VASc									
Mean score (±SD)	2.1 (±1.1)		2.7 (±1.4)		2.1 (±1.1)		*0.001*	*0.001*	0.837
Score—no. (%)									
0 or 1	20 (29 %)		26 (19 %)		30 (30 %)		*0.009*	*0.003*	0.993
2	25 (37 %)		37 (27 %)		36 (36 %)				
3	17 (25 %)		36 (26 %)		24 (24 %)				
≥4	6 (9 %)		39 (28 %)		10 (10 %)				
HAS-BLED									
Mean score (±SD)	1.4 (±0.8)		1.7 (±0.8)		1.4 (±0.8)		*0.001*	*0.003*	0.496
Score—no. (%)									
0 or 1	43 (63 %)		48 (35 %)		54 (54 %)		*0.001*	*0.011*	0.332
2	19 (28 %)		73 (53 %)		39 (39 %)				
≥3	6 (9 %)		17 (12 %)		7 (7 %)				
LAA morphology—no. (%)									
Banded LAA (chicken wings)	37 (54 %)		92 (67 %)		81 (81 %)		*0.002*	*0.005*	<*0.001*
Non-banded LAA (wind socks, cauliflower, cactus)	31 (46 %)		35 (25 %)		19 (19 %)				
Unknown	0 (0 %)		11 (8 %)		0 (0 %)				
Medications at time of inclusion—no. (%)									
ACE inhibitor/ARB	41 (60 %)		99 (72 %)		68 (68 %)		0.113	0.568	0.327
Amiodarone	4 (6 %)		21 (15 %)		9 (9 %)		0.069	0.171	0.564
Aspirin	14 (21 %)		15 (11 %)		10 (10 %)		0.087	1	0.072
Beta blocker	61 (90 %)		123 (89 %)		97 (97 %)		1	0.092	*0.026*
Calcium antagonist	8 (12 %)		35 (25 %)		18 (18 %)		*0.028*	0.208	0.385
Clopidogrel	0 (0 %)		3 (2 %)		0 (0 %)		0.552	0.266	1
Cardiac glycosides	7 (10 %)		16 (12 %)		19 (19 %)		1	0.138	0.136
Dronedarone	2 (3 %)		19 (14 %)		2 (2 %)		*0.014*	*0.001*	1
Statin	32 (47 %)		65 (47 %)		33 (33 %)		1	0.077	*0.033*
NSAR	16 (24 %)		15 (11 %)		9 (9 %)		*0.022*	0.67	*0.014*
PPI	35 (51 %)		67 (49 %)		42 (42 %)		0.767	0.357	0.27

*ACE* angiotensin-converting enzyme, *ARB* angiotensin receptor blocker, *LA* left atrium, *LAA* left atrial appendage, *LVEF* left ventricular ejection fraction, *NSAR* non-steroidal anti-rheumatic agents, *PPI* proton-pump inhibitor, *SD* standard deviation, *VKA* vitamin K antagonists

### Frequency of LA abnormalities

The groups of patients receiving VKA, dabigatran, and rivaroxaban medication did not yield significant differences with respect to frequency of LA abnormalities (Table 2). In summary, the frequency of LA abnormalities was lowest within the dabigatran group (3 %), followed by the rivaroxaban (5 %) and VKA group (9 %). A dense SEC (VKA: 1 %, dabigatran: 1 %, rivaroxaban: 2 %) was observed less frequently than a LA/LAA thrombus (VKA: 4 %, dabigatran: 0 %, rivaroxaban: 2 %), and a low LAAV of less than 20 cm/s (VKA: 4 %, dabigatran: 1 %, rivaroxaban: 1 %). The influence of VKA, dabigatran, and rivaroxaban on the risk of any LA abnormality was additionally analyzed by means of logistic regression models in order to prevent biased results due to imbalanced patient’s baseline characteristics. The results of the univariate and multivariate models are given in Table 3. The univariate models suggest that patients with CHADS_2_ score 2 and CHA_2_DS_2_-VASc score ≥4 have a significantly higher risk of any LA abnormality than patients with CHADS_2_-score 0–1 (OR 4.40, 95 % CI 1.54–12.54, *p* = 0.006) and CHA_2_DS_2_-VASc score 0–1 (OR 6.30, 95 % CI 1.28–30.95, *p* = 0.023). The multivariate model includes the CHADS_2_ score but not the CHA_2_DS_2_-VASc score. It suggests that patients with CHADS_2_ score 2 have a significantly higher risk of any LA abnormality than patients with CHADS_2_ score 0–1 (OR 4.07, 95 % CI 1.42–11.69, *p* = 0.009). Neither the univariate models nor the multivariate model indicate a significant difference between dabigatran and VKA and between rivaroxaban and VKA.

**Table 2:** Frequencies of LA abnormalities in the study population

	VKA, *n* = 138 (45 %)		Dabigatran, *n* = 68 (22 %)		Rivaroxaban, *n* = 100 (33 %)		*p* value for difference between VKA and Dabigatran	*p* value for difference between VKA and Rivaroxaban	*p*value for difference between dabigatran and rivaroxaban
LA/LAA thrombus	5 (4 %)		0 (0 %)		2 (2 %)		0.173	0.515	0.702
Dense SEC	2 (1 %)		1 (1 %)		2 (2 %)		1	1	1
LAAV <20 cm/s	5 (4 %)		1 (1 %)		1 (1 %)		0.666	1	0.405
LA abnormality	12 (9 %)		2 (3 %)		5 (5 %)		0.15	0.702	0.318

*LA* left atrium, *LAA* left atrial appendage, *LAAV* left atrial appendage velocity, *SEC* spontaneous echo contrast, *VKA* vitamin K antagonists

**Table 3:** Univariate and multivariate logistic regression analyses of risk factors of any LA abnormality

	Separate univariate models		Multivariate model^a^	
Clinical variable	OR (95 % CI)	*p* value	OR (95 % CI)	*p* value
Dabigatran vs. VKA	0.32 (0.07–1.46)	0.141	0.40 (0.08–1.88)	0.245
Rivaroxaban vs. VKA	0.55 (0.19–1.62)	0.28	0.65 (0.22–1.98)	0.453
CHADS2: 2 vs. 0–1	4.40 (1.54–12.54)	*0.006*	4.07 (1.42–11.69)	0.009
CHADS2: ≥3 vs. 0–1	3.80 (0.89–16.16)	0.071	3.19 (0.73–13.99)	0.124
CHA2DS2-VASC: 2 vs. 0–1	1.99 (0.38–10.55)	0.419		
CHA2DS2-VASC: 3 vs. 0–1	2.03 (0.36–11.41)	0.423		
CHA2DS2-VASC: ≥4 vs. 0–1	6.30 (1.28–30.95)	*0.023*		
HAS–BLED: 2 vs. 0–1	2.57 (0.87–7.60)	0.089		
HAS–BLED: ≥3 vs. 0–1	3.11 (0.70–13.80)	0.135		
Non-banded LAA vs. banded LAA	0.64 (0.21–1.99)	0.443		
Unknown LAA vs. banded LAA	0.00 (0.00,∞)	0.99		
Beta blocker	1.64 (0.21–12.84)	0.636		
Calcium blocker	0.21 (0.03–1.61)	0.133		
Dronedarone	1.49 (0.32–6.89)	0.61		
Statin	1.24 (0.49–3.13)	0.657		
NSAR	0.77 (0.17–3.47)	0.735		

*CI* confidence interval, *LAA* left atrial appendage, *NSAR* non-steroidal anti-rheumatic agents, *OR* odds ratio, *VKA* vitamin K antagonists

^a^Final model resulting from an all-subset variable selection based on Akaike’s Information Criterion; the medication group (dabigatran vs. VKA and rivaroxaban vs. VKA) was defined as fixed covariate, and the significant variables in column 1 were considered as possible covariates

## Discussion

In the present study, we investigated the frequency of three echocardiographic risk factors for stroke and systemic embolism in patients treated with either dabigatran and rivaroxaban, or with VKAs. Both NOACs showed numerically lower statistically non-different results in comparison to VKA for prevention of LA abnormalities in a low- to mid-risk cohort.

The current European and American guidelines recommend in AF >48 h either a sufficient therapeutic anticoagulation (INR >2) for at least 3 weeks or TEE prior to cardioversion to exclude LAA thrombus. Interestingly, the guidelines do not clearly discriminate between oral anticoagulation with VKAs and NOACs [9, 10]. This recommendation is based on results of subgroup analyses of the RE-LY and ROCKET-AF as well as the separate X-VeRT trial [13–15]. A recommendation regarding potential comparability of these different therapeutic anticoagulation regimens is based on subgroup analyses of the RE-LY as well as the ROCKET-AF trial. In the RE-LY trial, a cardioversion was performed in 1270 patients with AF. Stroke or systemic embolism at 30 days after cardioversion occurred in 0.8, 0.3, and 0.6 % of patients receiving dabigatran in a dose of 110 mg BID, 150 mg BID, and VKA, respectively [14]. In the ROCKET-AF trial, a cardioversion or catheter ablation was carried out in 321 patients. The incidence of stroke or systemic embolism at 30 days after cardioversion or ablation was 1.88 % in the rivaroxaban group and 1.86 % in the VKA group [15]. However, due to a markedly higher CHADS_2_ score, different study designs, and differences between TTRs, the event rates of the two NOACs could not be compared with each other. TTR in Rocket-AF was 55 % which was rather low and 64 % in the RE-LY study. A study published by Zylla and coworkers demonstrated in a high-risk population with a median CHA_2_DS_2_-VASc score of 4 that the prevalence of intracardiac thrombi under phenprocoumon was significantly higher (17.8 %) than in the dabigatran (3.8 %) or rivaroxaban group (4.1 %). Data about the TTR of VKA patients in which study are not available. Interestingly, subgroup analyses of the Rocket-AF data demonstrated that in rivaroxaban patients, the hazard ratio for the primary endpoint was above 1 when being compared with patients with a TTR of >67 %. Thus, sufficient VKA treatment may, despite other hurdles such as dependence of INR on oral vitamin K supply, higher interaction risk, and need for regular INR control, be comparable with NOACs as long as the TTR is high enough. This is especially important in patients where thromboembolic risk is per se not high. In our patients, TTR was 67 %. Despite clear trends that favor NOACs, we did not find significant differences between the two groups, even not with univariate or multivariate statistical models. Nevertheless, the results from the Zylla group imply that based on the relatively high incidence of LAA thrombi in a high-risk patient cohort, especially those under VKA, treatment may underlie a high risk of thromboembolic events during or after electrical cardioversion.

TEE is a moderately invasive method that allows a detailed evaluation of the structure and function of the LAA. It is accurate and the gold standard for identifying or excluding LA/LAA thrombus [29].

The incidence of LA/LAA thrombus under treatment with VKA depends on the patient population studied and different TTR values ranging from 1.5 to 17 % [30–33]. In most studies, however, the incidence of LA/LAA thrombus was between 2 and 7 % [30, 32]. Thus, the present data (4 % in the VKA group) are in line with these studies. In the RE-LY trial, the rate of LA/LAA thrombus was 1.8, 1.2, and 1.1 % in the dabigatran group of with 110 mg BID, 150 mg BID, and VKA group, respectively [14]. Notably, we did not observe any thrombus in the dabigatran group. The rivaroxaban group showed a numerically slightly higher incidence of LA abnormalities than the dabigatran group, which was, however, statistically not significant.

It has been shown that patients with AF and dense SEC have a likelihood of stroke of 22 %/year, despite anticoagulation [21]. Dense SEC is described with a frequency of 8–35 % in patients with AF, depending on the patient population, type and effectiveness of anticoagulation, and CHADS_2_-/CHA_2_DS_2_-VASc Score [19, 34–36]. Our results showed a relatively lower frequency of dense SEC (VKA: 1 %, dabigatran 1 %, rivaroxaban 2 %). A possible explanation for this difference is the high inter- and intraobserver variability in the grading of SEC [37].

Another important indicator for thromboembolic complications in patients with AF is the LAAV [34]. Reduction of the LAAV below 20 cm/s exhibited the highest association with stroke [38]. A LAAV less than 20 cm/s is found in 1 to 13 % of patients with AF [21, 34]. We observed an incidence of LAAV <20 cm/s that were numerically higher in patients receiving VKA (4 %) compared to patients receiving a NOAC (1 % for dabigatran and rivaroxaban). This observation is in line with the higher CHA_2_DS_2_-VASc score in this group.

It has been demonstrated that non-banded LAA is associated with lower LAAV and a higher frequency of SEC compared with banded LAA [39]. We found no significant association between the incidence of LA abnormalities and different LAA morphologies. Differences in the duration of AF/AFL in the different study populations could have, at least in part, contributed to these discrepancies.

Diastolic dysfunction is associated with a reduced LAA velocity, rate of SEC, and LAA thrombus in patient with non-valvular AF [40]. The impact of the three anticoagulatory drugs on LAA velocity, rate of SEC, and LAA thrombus in patients with non-valvular AF and diastolic dysfunction is unknown. Further studies are necessary to demonstrate the association between these parameters and LAA thrombus formation in patients with non-valvular AF under treatment with VKA and NOACs.

Several limitations of this study deserve attention. First, the number of investigated patients was relatively small. Second, there are further risk factors for stroke or systemic embolism in patients with AF, e.g., aortic plaques [19], which have not been addressed in the current trial. Third, we did not study patients being treated with Apixaban or edoxaban. Further studies should address the present approach in a larger patient cohort including apixaban and edoxaban patients. Fourth, the CHADS_2_/CHA_2_DS_2_-VASc scores are clinical classifications for predicting stroke in non-valvular AF and correlated with both dense SEC and LA/LAA thrombus [35]. The multivariate model in the present work also shows a close correlation between the CHADS_2_/CHA_2_DS_2_-VASc scores and LA-abnormalities. It is possible that the LA abnormalities are associated more with both scores than the anticoagulation drug.

## Conclusion

This is the first study addressing the incidence of LA/LAA thrombus formation in a predominantly low- to mid-risk patients cohort being treated with dabigatran or rivaroxaban compared with VKAs at a TTR of >65 %. This study demonstrates that with respect to the incidence of echocardiographic risk factors for thromboembolic events, both dabigatran and rivaroxaban are not inferior to VKA.

## Abbreviations

AF: atrial fibrillation
CHADS_2_: congestive heart failure, hypertension, age ≥75, diabetes mellitus, and prior stroke or transient ischemic attack (2 points)
CHA_2_DS_2_-VASc: congestive heart failure, hypertension, age (≥75: 2 points, 65–74: 1 point), diabetes mellitus, prior stroke or transient ischemic attack (2 points), vascular disease, sex category (female gender)
HAS-BLED: hypertension, abnormal renal or liver function, prior stroke, bleeding, labile INRs, elderly (age ≥65), drug therapy or alcohol intake
LA: left atrium
LAA: left atrial appendage
LAAV: left atrial appendage velocity
NOAC: non-vitamin K antagonist oral anticoagulants
SEC: spontaneous echo contrast
VKA: vitamin K antagonists
